# Editorial: Structural and functional venomics as a powerful approach for drug discovery and development

**DOI:** 10.3389/fphar.2024.1405681

**Published:** 2024-04-10

**Authors:** Mohamed A. Abdel-Rahman, Zhijian Cao, Tarek Mohamed Abd El-Aziz, Peter N. Strong, Jean-Marc Sabatier

**Affiliations:** ^1^ Zoology Department, Faculty of Science, Suez Canal University, Ismailia, Egypt; ^2^ National “111” Center for Cellular Regulation and Molecular Pharmaceutics, Key Laboratory of Fermentation Engineering (Ministry of Education), School of Life and Health Sciences, Hubei University of Technology, Wuhan, China; ^3^ Department of Internal Medicine, Cardiovascular Division, Washington University School of Medicine, St. Louis, MO, United States; ^4^ Zoology Department, Faculty of Science, Minia University, El-Minia, Egypt; ^5^ Biomolecular Sciences Research Centre, Sheffield Hallam University, Sheffield, United Kingdom; ^6^ Aix-Marseille University, CNRS, INP, Marseille, France

**Keywords:** animal venoms, venome peptidome, venome gland transcriptome, toxinome, venomics, antivenomics, drug discovery

The diversity of terrestrial and marine venomous creatures is extremely high with more than 200,000 species spread throughout the Animal Kingdom, including vertebrate animals (reptiles and fishes) and invertebrate animals (marine cone snails, octopi, leeches, myriapods, insects and arachnids). Venomous animals have a specialized tissue or organ called the “venom gland” in which their venoms are produced/stored and introduced into the prey by a specialized delivery apparatus such as a fang, stinger, teeth, or harpoon. Animal venoms are very complicated and often deadly cocktails, which contain unique mixtures of toxins (mainly peptides and proteins) targeting vital systems of the victim or prey. For their high degree of specificity and potency, venom peptides have been used as good templates to develop various venom-derived drugs such as Captopril, Ziconotide, and Exenatide for treating hypertension, chronic pain, and type-2 diabetes, respectively. Also, the scorpion venom peptide chlorotoxin (ClTx) is a useful tumor imaging agent and radiolabelled ClTx has undergone clinical trials for targeted glioma radiotherapy.

Generally, the application of cutting-edge “OMICS” technologies in venom research (venomics to include proteomics, transcriptomics and high-throughput functional assays) is extremely advantageous for several reasons: (1) gaining a better understanding of venom composition and evolution, (2) revealing molecular mechanisms involved in venom toxicity and developing effective antivenoms, (3) understanding biological processes taking place in venom glands and (4) discovery of novel diagnostic and pharmacological agents that can be used in research and medicine. Accordingly, this Research Topic mainly focuses on the new structural and functional findings (with biological and pharmacological significance) obtained from the venom of terrestrial and marine venomous animals with the aid of venomics technology. Our Research Topic of Frontiers in Pharmacology includes five original research manuscripts discussing the significance of venomics approaches in the structural and functional characterization of various toxins (α-Elapitoxin-Nn2a and α-Elapitoxin-Nn3a; apamin; omega-lycotoxin-Gsp2671e; μ-Conotoxin KIIIA and αD-conotoxins VxXXB) derived from snake (*Naja naja*), bee (*Apis mellifera*), spider (*Lycosa praegrandis*), and marine *Conus* (*Conus kinoshitai and Conus vexillum*) venoms, respectively.

In the first article described by Huynh et al., two postsynaptic neurotoxins, α-Elapitoxin-Nn2a and α-Elapitoxin-Nn3a, were isolated and functionally characterized from the venom of Indian Cobra (*Naja naja*) using a combination of proteomic and pharmacologic approaches. Structural analyses revealed that α-Elapitoxin-Nn2a has 62 residues (7,020 Da with four disulfide bridges) and belongs to short-chain neurotoxins. On the other hand, α-Elapitoxin-Nn3a has 71 residues (7,807.5 Da with five disulfide bridges) and belongs to long-chain neurotoxins. Using chick biventer cervicis nerve-muscle preparation, both toxins induced concentration-dependent inhibition of indirect twitches and reduction in contractile responses to exogenous nicotinic agonists (acetylcholine and carbachol). More importantly, the *in vitro* addition of Indian polyvalent antivenom can reverse the neurotoxicity induced by the long-chain neurotoxin Nn3a, but not the short-chain neurotoxin Nn2a.

The second article (Kuzmenkov et al.) of this Research Topic discusses the contradiction in the structural and electrophysiological data of an important neurotoxin derived from insect venom. In the 1960s, a small peptide (18 amino acid residues, 2026.7 Da) called apamin was purified from the honeybee *A. mellifera* venom. When injected into mice, it was shown to induce muscle spasms, jerks, and convulsions. Apamin selectively acts on small conductance Ca^2+^-activated potassium channels (KCa^2^). However, there is a controversy regarding the publications discussing structural and pharmacological data of apamin. Through extensive structural and electrophysiological profiling of this toxin against several molecular targets (five Ca^2+^-activated K^+^ Channels (KCa), one inwardly rectifying K^+^ channels (Kir), 15 Voltage-gated K^+^ channels (Kv), 10 Voltage-gated Na^+^ channels (Nav), three Acid-sensing ion channels (ASIC), one transient receptor potential channels (TRP), three glutamate receptors (GluR) and three nicotinic acetylcholine receptors (nAChR)), the contradicting claims have been precisely reconciled in the Kuzmenkov et al. article. The NMR spectroscopy data showed that apamin forms a β-turn (Asn2–Ala5) and a short ɑ-helix (Ala9–Gln16) and that there are no electrostatic, π-cation, or stacking interactions that might sustain the observed apamin structure. The findings show that apamin selectively inhibits KCa^2^ channels (KCa^2.1^, KCa^2.2^, and KCa^2.3^) at nanomolar or sub-nanomolar concentrations (IC_50_ values were 4.1 nM, 87.7 p.m., and 2.3 nM, respectively), with negligible impact on other molecular targets. Accordingly, the structural and functional data presented in this work support the utilization of apamin as a KCa2-selective pharmacological tool and a good model for developing new drugs.

The third article in this Research Topic examined how omega-lycotoxin-Gsp2671e (as a P/Q-type VGCC modulator) from *L. praegrandis* spider venom improves memory in glutamate-induced excitotoxicity rats (Keimasi et al.). Various venomics and behavioral analyses were used in this investigation including venom extraction, gel filtration chromatography, capillary electrophoresis, mass spectrometry, acute cytotoxicity (LD_50_), Morris Water Maze task, and Novel Object Recognition test (to assess long and short-term memory). In hyper-stimulated NMDA (N-Methyl-D-aspartate receptors) rats, OLG1e administration resulted in increased expression of synaptophysin (SYN), synaptosomal-associated protein-25 kDa (SNAP-25), and synaptotagmin 1 (SYT1), as well as the restoration of upper levels of field excitatory postsynaptic potentials (fEPSP) in comparison to the non-treated NMDA group. The results of this study can be used as a foundation for future analyses of how OLG1e affects cognitive functions like pain sensitivity, associative learning, sensorimotor and locomotor ability.

The fourth (Kimball et al.) and fifth (Ho et al.) articles of this Research Topic discuss the electrophysiological activity of two important conotoxins (μ-conotoxin KIIIA and αD-VxXXB, respectively) derived from the venoms of marine Conus. Kimball and colleagues used a combination of *in silico* (Rosetta Computational Modeling and Rosetta Dock) and experimental approaches to investigate the molecular interaction between μ-conotoxin KIIIA (KIIIA) and human Nav1.7 channel (hNav1.7). The obtained results through this integrative approach can be potentially useful for developing specific peptide-based therapeutics targeting Nav1.7 which plays a crucial role in pain signaling. Moving to the second conotoxin αD-VxXXB (Ho et al.) which is derived from the venom of *C. vexillum.* Through a non-competitive (allosteric) mechanism, αD-conotoxins (11 kDa homodimers) potently block nicotinic acetylcholine receptors (nAChRs). Ho and colleagues described the allosteric binding mechanism between the extracellular ligand-binding domain of nAChRs and the granulin-like C-terminal (CTD) of VxXXB. According to mutational and docking studies, the CTDs of αD-VxXXB bind cooperatively at two unique allosteric sites on nAChRs. Thus, the information that Ho and colleagues have acquired opens up a new direction in the development of novel subtype-specific allosteric nAChR antagonists.

Finally, the research findings presented through this Research Topic clearly demonstrate the usefulness of venomics approaches in venom separation, identification, and characterization of various venomous animals. Different venom peptides have been characterized and can be considered good templates for developing effective candidate drugs targeting various diseases. We are confident that this Research Topic of articles will motivate several toxinologists and clinicians globally to do further research in the field of natural toxins derived from venomous animals using the cutting-edge technologies of venomics.

